# Low diagnostic accuracy of Xpert MTB/RIF assay for extrapulmonary tuberculosis: A multicenter surveillance

**DOI:** 10.1038/s41598-019-55112-y

**Published:** 2019-12-06

**Authors:** Mohammadreza Allahyartorkaman, Mehdi Mirsaeidi, Gholamreza Hamzehloo, Sirus Amini, Mona Zakiloo, Mohammad Javad Nasiri

**Affiliations:** 1 Regional Tuberculosis Reference Laboratory, Tehran University of Medical Sciences, Tehran, Iran; 2 Department of Medicine, Division of Pulmonary, Critical Care, Sleep and Allergy, University of Miami, Coral Gables, Florida USA; 3 Department of Microbiology, School of Medicine, Shahid Beheshti University of Medical Sciences, Tehran, Iran

**Keywords:** Infectious-disease diagnostics, Tuberculosis

## Abstract

Diagnostic accuracy of Xpert MTB/RIF assay for pulmonary tuberculosis (PTB) and extrapulmonary TB (EPTB) has not been investigated in Iran. This study was aimed to assess the diagnostic accuracy of Xpert MTB/RIF assay for both PTB and EPTB. A total of 2111 clinical samples (1218 pulmonary and 838 extra-pulmonary) were collected from 16 medical centers during the study period and were analyzed for detection of PTB and EPTB by both Xpert MTB/RIF assay and standard conventional methods (culture and direct smear microscopy). The sensitivity, specificity, positive predictive value (PPV) and negative predictive value (NPV) of Xpert MTB/RIF assay for PTB were found to be 95.5%, 96.7%, 83.8%, and 99.1% respectively. For EPTB, the sensitivity, specificity, PPV and NPV of Xpert MTB/RIF assay counted for 76.5%, 95.9%, 62%, and 97.9% respectively. Xpert MTB/RIF assay found to be highly sensitive, specific and comparable to standard conventional methods for the diagnosis of PTB. However, the sensitivity and specificity of Xpert MTB/RIF for EPTB specimens were highly variable; thus, Xpert MTB/RIF cannot be recommended to replace standard conventional tests for diagnosis of EPTB.

## Introduction

Tuberculosis (TB) still remains a public health problem with an increasing death rate worldwide, especially in developing countries^1^. Early detection of bacilli in clinical samples and starting sufficient treatment is extremely important to reduce the death rate^2,3^. Acid-fast bacilli microscopy and culture are still the cornerstones of TB diagnosis^4^. Although culture is a gold standard technique but is cumbersome and time-consuming^5,6^. Likewise, the microscopy, although rapid and inexpensive, but its sensitivity is variable (20–80%)^6^. In the context of these limitations, Xpert MTB/RIF assay, a fully automated real-time semi-nested PCR system was endorsed by World Health Organization (WHO) as the most rapid test for diagnosis of pulmonary TB (PTB)^7^. Extrapulmonary TB (EPTB) accounts for more than 20% of all TB cases, and even higher percentages in HIV-infected persons^1,8^. Differently from PTB, the diagnosis of EPTB is still a serious problem and existing tests are limited in accuracy^8^. Given the limitations of tests for EPTB detection, Xpert MTB/RIF has been evaluated in several studies^9–15^. Although Xpert MTB/RIF assay has been validated for TB detection in sputum by authors in different countries, the efficacy of this automated molecular platform for TB detection in nonrespiratory specimens was highly variable, as its sensitivity ranged from 30% to 100%^16–26^. Furthermore, the efficacy of Xpert MTB/RIF has not been yet investigated in Iran. Thus, the current study was aimed to comprehensively analyze the diagnostic accuracy of Xpert MTB/RIF assay for PTB and EPTB in Iran.

## Methods

### Setting and sampling

This cross-sectional study performed from Sep 2015 to Jan 2018 in regional reference laboratory of TB in Tehran, Iran. This center is well-equipped biosafety level III laboratory facilities, and standard biosafety precautions were followed for specimen processing, inoculation, and drug susceptibility testing. The Swedish Institute for Infectious Disease Control monitored and supervised the laboratories’ quality. A total of 2111 specimens from TB suspected cases from 6 public hospitals and 10 medical universities from different provinces of Iran were included in this study. From the collected samples, 1218 (59.2 percent) were pulmonary; bronchoalveolar lavage fluid (BAL), tracheal, sputum and 838 (40.7 percent) were extrapulmonary; urine, abscess, osteoarticular, biopsy, pericardium, cerebrospinal fluid (CSF), gastric lavage, blood, real-time fluid, and ascites.

Specimens were collected from both outpatients and inpatients with a history of cough, unintentional weight loss, and fever for more than two weeks. Specimens were either from new cases or from patients with treatment failure or relapse.

The Ethics Committee of Shahid Beheshti University of Medical Sciences, Tehran, Iran approved the study and all research was performed in accordance with the relevant guidelines. All the patients and/or their legal guardians have signed informed consent.

### Microscopy examination and culture

Specimens (2.5 to 10 mL) were decontaminated using 4% sodium hydroxide (NaOH) (Petroff method)^27^. Sediments of each treated sample were used to prepare a smear for Ziehl-Neelsen and Auramine fluorochrome staining and were cultured in Löwenstein-Jensen medium^27,28^. Only one culture isolated per study subject was considered for further analysis. Each isolate was examined regarding morphology, pigmentation, and date of growth. Bacterial isolates identified as *M. tuberculosis* using standard biochemical tests, including production of niacin, nitrate reduction and catalase^27^.

### Xpert MTB/RIF

One mL unconcentrated specimens used (without centrifuge) for Xpert MTB/RIF assay. Specimens, firstly, homogenized with a 5% acid-alcohol buffer, a 2:1 ratio as the Cepheid Company (USA) prepared in sample reagent (SR) and then added to the Xpert MTB/RIF cartridge to do the assay in Genexpert instrument. Xpert MTB/RIF, also, uses five overlapping probes (A, B, C, D, and E) to detect five mutations in the *rpoB* gene that accord rifampicin resistance^2^.

### Data analysis

The sensitivity, specificity, positive predictive value (PPV) and negative predictive value (NPV) with 95% confidence intervals were calculated for the Xpert MTB/RIF, using culture and direct smear microscopy as the gold standard. The summary receiver operating characteristic (SROC) curve was constructed based on a bivariate regression approach to show the level of accuracy of the Xpert MTB/RIF. The multiple regression analysis was used to shows any relationship between semi-quantitative Xpert MTB/RIF results and direct smear and culture results. All statistical analyses were performed with MedCalc (version 14.8.1, Medcalc Software).

## Results

### Microbiological findings

As shown in Table 1, of 1218 pulmonary specimens, 171 were positive and 982 were negative by standard conventional methods. Likewise, of 838 extrapulmonary specimens, 49 positive and 713 were negative by standard methods. Biopsy (26.5%), abscess (20.4%) and pleural fluid (14.2%) were the most commonly involved organs for EPTB.

**Table 1:** Xpert MTB/RIF assay performance for pulmonary and extrapulmonary specimens.

Category	Specimen type	Number (%)	Xpert MTB/RIF Error& Invalid	True negative	False negative	True positive	False positive	Xpert Sensitivity (%) with 95% CI	Xpert Specificity (%) with 95% CI	Xpert Positive Predictive Value (%) with 95% CI	Xpert Negative Predictive Value (%) with 95% CI
Pulmonary	BAL	340 (16.5)	4	327	0	3	6	100 (29.2–100)	98.2 (29.2–100)	33.3 (18.4–52.4)	100
	Tracheal	30 (1.4)	0	29	0	1	0	100 (25–100)	100 (88–100)	100	100
	Sputum	848 (41.2)	20	626	8	175	19	95.6 (91.5–98)	97 (95.4–98.2)	90.2 (85.5–93.4)	98.7 (97.5–99.3)
Extrapulmonary	Urine	44 (2.1)	2	40	0	2	1	100 (15.8–100)	97.5 (86.8–99.9)	66.6 (22.4–93.2)	100
	Abscess	49 (2.4)	1	36	0	10	2	100 (69.1–100)	94.7 (82.2–99.3)	83.3 (56.4–95)	100
	Osteoarticular	80 (3.4)	2	66	2	3	7	60 (14.6–94.7)	90.4 (81.2–96)	30 (13.5–53.9)	97 (91.8–98.9)
	Biopsy	162 (7.8)	9	119	5	13	16	72.2 (46.5–90.3)	88.1 (81.4–93)	44.8 (32–58.2)	95.9 (91.8–98)
	Pericardium	120 (5.8)	2	112	3	2	1	40 (5.2–85.3)	99.1 (95.1–99.9)	66.6 (17.7–94.8)	97.3 (94.8–98.7)
	Cerebrospinal fluid (CSF)	44 (2.1)	1	39	0	2	1	100 (15.8–100)	97.5 (86.8–99.9)	66.6 (22.4–93.2)	100
	Gastric Lavage	111 (5.4)	2	103	1	4	1	80 (28.3–99.4)	99 (94.7–99.9)	80 (35.1–96.7)	99 (94.6–99.8)
	Blood	15 (0.7)	2	13	0	0	0	—	100 (75.2–100)	—	100
	Plural fluid	175 (8.4)	7	153	4	7	4	63.6 (30.7–89)	97.4 (93.6–99.3)	63.6 (37.6–83.5)	97.4 (94.5–98.8)
	Ascites	38 (1.8)	2	33	0	3	0	100 (29.2–100)	100 (89.4–100)	100	100
Pulmonary		1218 (59.2)	24	982	8	179	25	95.7 (91.7–98.1)	97.5 (96.3–98.3)	87.7 (82.9–91.3)	99.1 (98.4–99.5)
Extrapulmonary		838 (40.7)	31	713	15	49	30	76.5 (64.3–86.2)	95.9 (94.2–97.2)	62 (52.8–70.4)	97.9 (96.8–98.6)
Total Number		2056	55	—	—	—	—	—	—	—	—

### Xpert MTB/RIF results

*M. tuberculosis* was present in 204 (13.4%) of pulmonary specimens and 79 of extrapulmonary specimens (Table 1). “Invalid,” “error,” and “no result” of Xpert MTB/RIF were excluded from the study. The proportion of Xpert MTB/RIF invalid and error cases in extrapulmonary specimens was significantly more than pulmonary specimens with Chi-squared 5.62 (*p* < 0.02). Comparison of false positives in pulmonary and extrapulmonary specimens have not shown meaningful difference whilst false-negative results illustrated a significant difference with Chi-squared 5.93 for two groups (*p* < 0.02). The percentage result for each subgroup specimen of pulmonary and extrapulmonary are showed in Table 1. Furthermore, 22 rifampicin resistance (7.7% of positive cases) and 13 rifampicin indeterminate (4.5% of positive cases) were also indicated by Xpert MTB/RIF.

### Sensitivity, specificity, PPV, and NPV

The overall sensitivity, specificity, PPV and NPV of Xpert MTB/RIF assay for PTB were found to be 95.5%, 96.7%, 83.8%, and 99.1% respectively. For EPTB, the sensitivity, specificity, PPV, and NPV of Xpert MTB/RIF assay were found to be 76.5%, 95.9%, 62%, and 97.9%, respectively.

**Figure 1:**
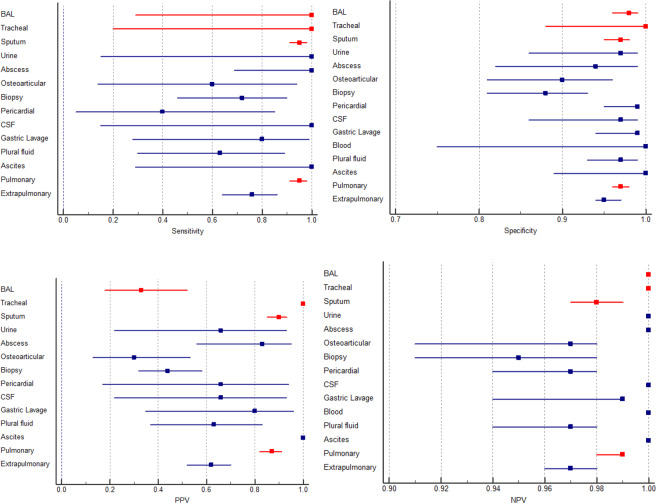
Forest plots give details of sensitivity, specificity, PPV, and NPV. BAL: bronchoalveolar lavage fluid, CSF: cerebrospinal fluid (Red colors are representative for pulmonary samples).

Among extrapulmonary specimens, osteoarticular and pericardial specimens have lower sensitivity rather than average EPTB sensitivity (*p* < 0.0001). A comparison of sensitivity between PTB and EPTB showed a significant difference with 95% and 76% respectively (*p* < 0.0001). With the same respect, NPV for both groups was very high, while comparison of PPV between PTB and EPTB revealed a significant difference (*p* < 0.0001) (Table 1). The forest plot (Fig. 1) shows the sensitivity, specificity, PPV, and NPV for each group of specimens. The inter-rater agreement test for PTB and EPTB showed Xpert MTB/RIF assay has substantial agreement (kappa 0.74) and moderate agreement (kappa 0.4) with standard conventional methods, respectively. The accuracy index for PTB and EPTB was 96% and 94%, respectively.

**Figure 2:**
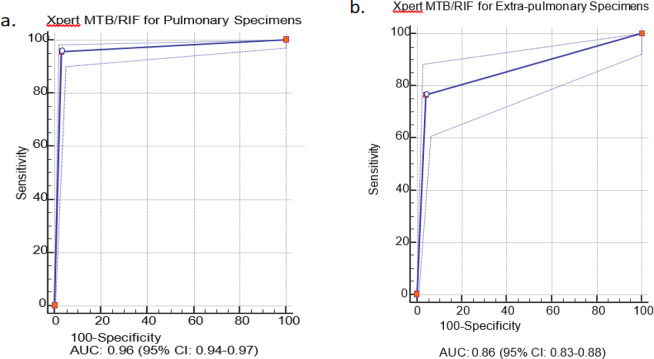
The summary receiver operating characteristic (SROC) curves for Xpert MTB/RIF assay. The SROC plot shows a summary of test performance, visual assessment of threshold effect, and heterogeneity of data in SROC space between sensitivity and specificity. The dashed blue line that is around the point estimate (blue line) shows 95% confidence region. The area under the curve (AUC), acts as an overall measure for test performance. Particularly, when AUC would be between 0.8 and 1, the accuracy is relatively high. As a matter of fact, AUC was 0.96 for pulmonary specimens in this report which represented a high level of accuracy. If SROC curve was in the upper left corner it would show the best combination of sensitivity and specificity for the diagnostic test. Part “a” and “b” are representative for pulmonary and extra-pulmonary specimens, respectively.

Based on the SROC curves (Fig. 2), the AUC for pulmonary and extrapulmonary specimens was 0.96 (95% CI: 0.94–0.97) and 0.86 (95% CI: 0.83–0.88), respectively (*p* < 0.0001).

### Regression analysis

**Figure 3:**
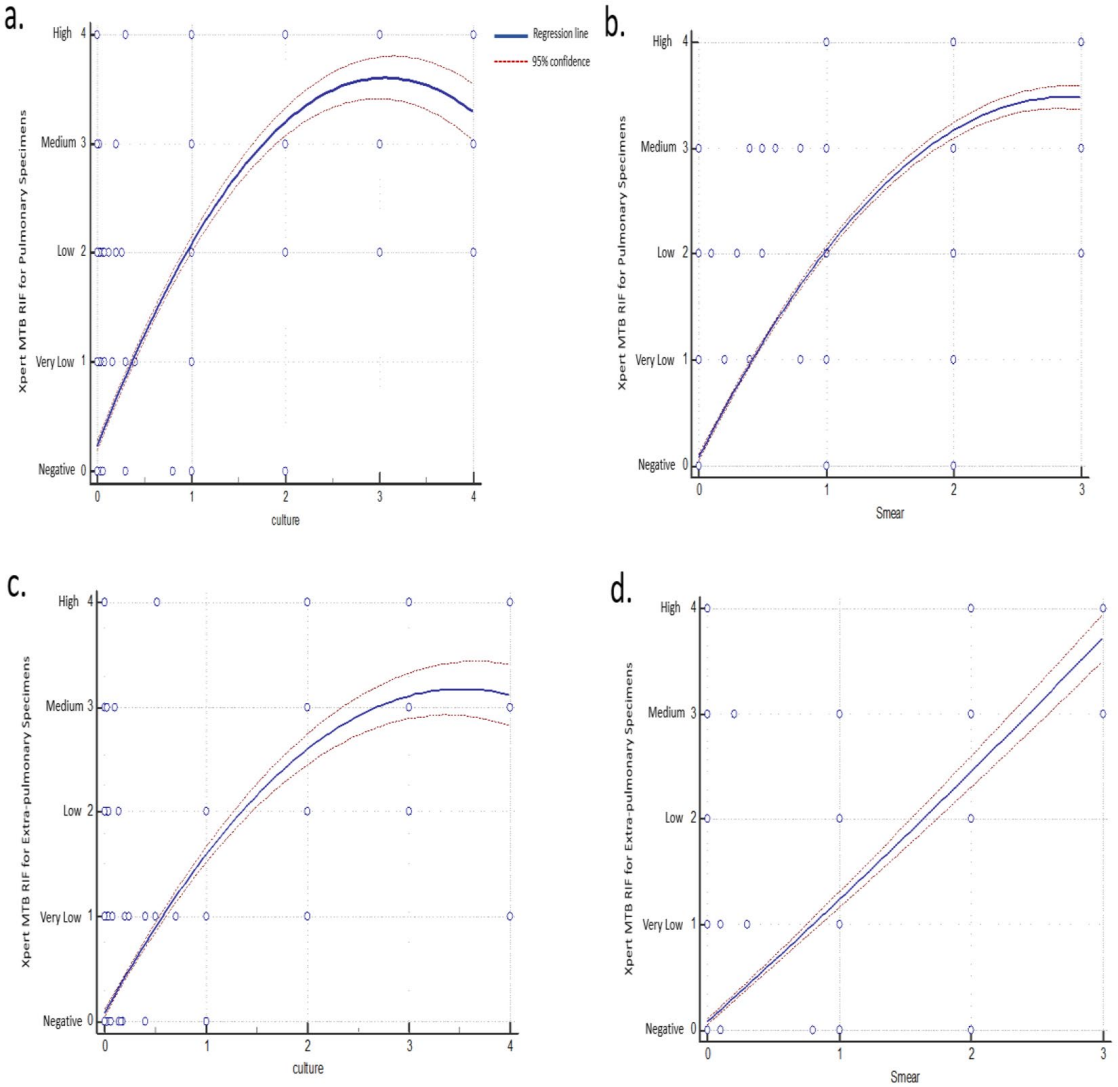
Quadratic regression plot to compare the Xpert MTB/RIF results with culture and smear microscopy. On Xpert MTB/RIF axis, results are categorized as 0, 1, 2, 3 and 4 which are indicated as negative, very low, low, medium and high scales, respectively. On the smear axis, results are categorized as 0, 1, 2 and 3 which are indicated as negative, 1+, 2+ and 3+ grading, respectively. On culture axis, results are categorized as 0, 1, 2, 3 and 4 which are indicated as negative, 1+, 2+, 3+ and 4+ grading, respectively. Part “a” and “b” are representative for pulmonary specimens and part “c” and “d” are representative for extra-pulmonary specimens. Regression lines showed the correlations between Xpert MTB/RIF results and culture/direct smear methods results.

Based on our regression analysis, Xpert MTB/RIF results were used as regressand (dependent variables) and culture/direct smear results were used as regressors (independent variables). According to the Xpert MTB/RIF assay, results are semi-quantitative and are categorized as “Very low,” “Low,” “Medium,” and “High” based on the levels of detection. Likewise, the results of conventional methods are also semi-quantitative, which could be categorized as 1+, 2+, and 3+ in direct smear assay, and 1+, 2+, 3+ and 4+ in culture assay. Thus, we were able to compute the relationships among these variables. Accordingly, as shown in Fig. 3, there were correlations between culture/direct smear results and Xpert MTB/RIF results. Therefore, we were able to assess the relationship between the results of Xpert MTB/RIF and culture/direct smear results.

## Discussion

This is the first assessment of Xpert MTB/RIF performance conducted on a large sample size in Iran. In the current study, sensitivity for pulmonary specimens was higher than in extrapulmonary samples (95.5% vs. 76.5%).

Similarly, several studies evaluated the accuracy of Xpert MTB/RIF on pulmonary specimens; with sensitivity varies from 95% to 100% in smear-positive sputa^29–34^.

Although, previous studies showed sufficient accuracy of Xpert MTB/RIF assay for diagnosis of PTB, an acceptable accuracy for EPTB has not been well established^22,23,35–37^. In the current study, Xpert has high specificity but limited sensitivity for the detection of TB in extrapulmonary specimens. Although positive results may be useful in rapidly identifying the disease, negative results provide less certainty for ruling out EPTB. The sensitivity of Xpert MTB/RIF for TB detection in extrapulmonary samples varied widely across different sample types explain more (From 40% to 100%) (Table 1). As reported by other studies, it is possible that the decontamination step has determined the lowering of bacillary load and consequently the reduction of test sensitivity^8,38^. In agreement with these findings, our study indicated that specimens, which need smashing and homogenization (i.e. osteoarticular and biopsy specimens), had lower sensitivity.

Xpert MTB/RIF assay is based on real-time bacon PCR and subsequently is sensitive to PCR inhibitors; therefore, it needs special protocols and/or treatment buffer for extrapulmonary specimens, which includes variety of specimens^23^. The current Xpert MTB/RIF buffer has been developed for sputum. This may affect the outcome with false-negative results and decrease the sensitivity for specimens other than sputum.

We found that Xpert MTB/RIF results for pericardial and biopsy specimens are less sensitive with low PPV, which was likely due to the presence of blood and inhibitors for PCR reaction. Therefore, any treatment to remove blood before the reaction would increase the sensitivity of these specimens. Low PPV for osteoarticular specimens was also an important issue that should be addressed with further investigation.

Based on regression analysis, we were able to assess the relationship between the results of Xpert MTB/RIF and culture/direct smear results for PTB and EPTB specimens.

### Limitation of the study

The current study is limited by a low sample size for blood and CSF specimens.

## Conclusions

Xpert MTB/RIF could be used as the first-line diagnostic tool for PTB cases. However, the overall low sensitivity of Xpert MTB/RIF for extrapulmonary specimens precludes the use of this test to rule out EPTB with certainty.
